# Characterization of Rubella Seronegative Females in the Zambian Blood Donor Community

**DOI:** 10.3389/fpubh.2015.00059

**Published:** 2015-04-24

**Authors:** Mazyanga L. Mazaba, Mwaka Monze, Olusegun A. Babaniyi, Seter Siziya, Charles Michelo

**Affiliations:** ^1^World Health Organization, Lusaka, Zambia; ^2^University Teaching Hospital, Lusaka, Zambia; ^3^Copperbelt University, Ndola, Zambia; ^4^University of Lusaka, Lusaka, Zambia; ^5^University of Zambia, Lusaka, Zambia

**Keywords:** rubella, seronegative, female, blood donors, Zambia

## Abstract

Rubella is an acute, contagious viral infection caused by a teratogenic enveloped single-stranded RNA virus, rubella virus, a member of the togaviridae family. Though causing generally mild infections in children and adults, it is a disease of public health importance in pregnant women causing major problems including abortions, miscarriages, and congenital rubella syndrome in more than 20% of the susceptible population. This study was carried out to determine the characteristics associated with rubella seronegativity among female blood donors in Zambia. Rubella-specific IgG antibody levels were measured in the blood serum. Proportions were compared using the Chi-squared test at the 5% significance level, and magnitudes of associations were determined using the odds ratio and its 95% confidence interval. Of the 124 female blood donors tested for rubella IgG 46.0% were aged <20 years. Overall, 66.7% of the participants had never been married. More than half (62.1%) of the participants resided in rural areas of the country. Of the 114 participants with recorded level of education, 50.1% had at least completed secondary school. Out of 43 participants with recorded current employment status, 44.2% were not working for pay. A total of 10 (8.1%) participants were seronegative to rubella IgG antibodies. No factors were associated with seronegativity. Protection against rubella through natural infection appears inadequate to protect the population, increasing the risk of CRS.

## Introduction

Rubella is an acute, contagious viral infection caused by a teratogenic enveloped single-stranded RNA virus, rubella virus, a member of the togaviridae family (1–3). Although the disease affects both males and females, it is, however, a disease of public health importance in pregnant women causing major problems including spontaneous abortions, miscarriages, and congenital defects including hearing impairment, heart defects, and cataracts [congenital rubella syndrome (CRS)] in more than 20% of the susceptible population (4–6). Rubella virus is the major cause of birth defects among the TORCH group of agents (*Toxoplasma*, Others, Rubella, Cytomegalo virus, and Herpes virus), which cause congenital anomalies (7).

Rubella is vaccine-preventable and the primary objective of rubella-control programs is prevention of congenital rubella virus infection, which includes CRS (8). Control and prevention of rubella infection is by immunization and therefore the World Health Organization (WHO) recommends administration of combined rubella and measles vaccines to young children, whenever feasible, to prevent CRS. Primary infection with rubella also leads to immunity (9). Various strategies have been implemented in different countries. Rubella immunization of adolescent girls and susceptible women contemplating pregnancy are additional control strategies. The United States (US) Centers for Disease Control reported that universal vaccination of young children and selective vaccination of susceptible women quickly resulted in the elimination of CRS cases in US (4) and Americas (9). Hinman and team suggested prioritizing protecting women of childbearing age first, and then interrupting transmission of rubella (10). Two live attenuated virus vaccines, RA 27/3 and Cendehill strains were effective in the prevention of adult disease. However, their use in prepubertile females did not produce a significant fall in the overall incidence rate of CRS. Reductions were only achieved by immunization of all children (11). The vaccine is now usually given as part of the MMR and MR vaccine. The WHO recommends that the first dose be given at 12–18 months of age with a second dose at 36 months (12).

Understanding the specific epidemiology of rubella in a country would help with determining the strategy to be used in prevention and control of rubella and CRS. Rubella and CRS epidemiology is poorly understood in most parts of the developing world including Zambia with gaps in the serosurvey literature of rubella regarding immunity across the entire age spectrum, among males and females, and in varying settings that must be addressed (13). A few seroprevalence studies have been conducted to determine the susceptibility of women of child bearing age to rubella in the developing world: a study to determine susceptibility to rubella among women of child bearing age revealed 95.5% seropositivity in Turkey (14); 90.1% in women 15–45 years of age in Senegal, and 84% in women 15–34 years of age in Côte d’Ivoire (15). Although it has already been established that rubella disease exists in Zambia (16) with a seroprevalence of 91.9% among female blood donors in Zambia (17), it is important to determine characteristics associated with rubella seronegativity of the immunoglobulin G (IgG) antibody among female blood donors aged 16 years or older in order to inform policy on rubella-control strategy that can be adopted for Zambia.

## Materials and Methods

The current study used data collected in a cross section study among blood donors in Zambia (17). Details of the methodology, which was used in that study, are highlighted below. Data were collected from all the nine provincial headquarters (18). The sample size was calculated using Statcalc program in Epi Info version 3.5.4 for population surveys and considering the following parameters: rubella IgM positive seroprevalence rate of 76.9% (19), a population size of 108,296, and an error of 5%, the required sample size was 307. Totals of 203 males and 124 females participated in the survey and only females were considered in the current study. The sample size was proportionally allocated to provinces. Because the total number of persons donating blood is small, all eligible persons were requested to participate in the survey.

Blood donors are mostly students (70%). The study questionnaire included the following items: age, sex, residency, economic status, education, reported rubella infection or vaccination, and rubella IgG result (positive/negative IgG in ELISA test). The questionnaire was pretested to ensure validity.

Blood samples were collected from consenting participants and sent to the provincial laboratory for serum separation and storage until transportation to the University Teaching Hospital, Virology laboratory, for testing using the Enzyme Linked Immunosorbent Assay (20). Anti-rubella-virus/IgG test result was considered negative if the difference in the optical densities between the antigen and antigen control wells was <0.100, positive if the difference was >0.200 and equivocal if the difference was from 0.100 to 0.200.

Epi Data version 3.1 was used to enter data and analysis was conducted using SPSS version 16.0. About 75% of the participants were aged below 25 years and in order to obtain valid statistical results in cross tabulations, the factor age was categorized into <20 years and 20 years or older age groups.

The Yates corrected Chi-squared test was used to determine associations between qualitative factors in 2 by 2 tables. Meanwhile, the Pearson’s Chi-squared test was used to test associations in contingency tables higher than 2 by 2. The cut off point for statistical significance was set at 5%. Magnitudes of association were estimated using odds ratios and their 95% confidence intervals.

The research protocol was approved by the University of Zambia Biomedical Research Ethics Committee Assurance No. FWA00000338, IRB00001121 of IORG0000774. Permission to conduct the study was obtained from the Zambia National Blood Transfusion Services and Ministry of Health. Before administering the questionnaire and obtaining a blood sample, study procedures were explained to participants and consent to participate in the survey was obtained. Participants were interviewed privately. Results were shared with the blood bank for further dissemination to their clients who participated in the survey and wished to know their serostatus to rubella IgG.

## Results

A total of 124 female blood donors were tested for rubella infection of which 46.0% were aged <20 years. Table 1 shows the distribution of socio-demographic and economic factors stratified by age group. Overall, 66.7% of the participants had never been married. More than half (62.1%) of the participants resided in rural areas of the country. Of the 114 participants with recorded level of education, 50.1% had at least completed secondary school level of education or higher. Out of 43 participants with recorded current employment status, 44.2% were not working for pay.

**Table 1:** **Distribution of participants by socio-demographic and economic factors stratified by age**.

Factor	Overall	Aged <20 years	Age 20+ years	*p* value
	*n* (%)	*n* (%)	*n* (%)	
**Marital status**
Never married	80 (64.5)	55 (96.5)	25 (37.3)	<0.001
Currently or once married	44 (35.5)	2 (3.5)	42 (62.7)	
**Residence**
Urban	47 (37.9)	34 (59.6)	13 (19.4)	<0.001
Rural	77 (62.1)	23 (40.4)	54 (80.6)	
**Education level**
No formal school/up to secondary school	63 (55.3)	26 (53.1)	37 (56.9)	0.826
Secondary school completed/college/pre-university or higher	51 (44.7)	23 (46.9)	28 (43.1)	
**Current employment**
Government/non-government/self employed/employer	24 (55.8)	6 (42.9)	18 (62.1)	0.389
Not working for pay	19 (44.2)	8 (57.1)	11 (37.9)	

**Figure 1:**
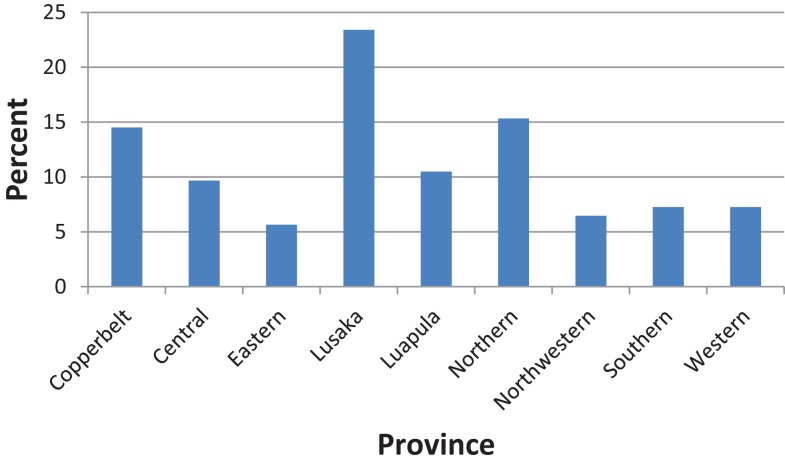
**Distribution of participants by province**.

About one in four (23.4%) of the participants resided in Lusaka province (Figure 1). Most of the participants (62.1%) resided in rural settings of Zambia.

A total of 10 (8.1%) participants were seronegative to rubella IgG antibodies. Of the factors considered in the analysis, none of them was significantly associated with rubella seronegative.

## Discussion

This study determined the characteristics associated with rubella seronegative among female blood donors in Zambia between 16 and older. This study reveals a rubella susceptible population (seronegative) of 8.1% among female blood donors in Zambia.

Although rubella has been eliminated in US, and in control in other parts of the developed world it continues to be endemic in many parts of the world. It is estimated that more than 100,000 infants are born with CRS annually worldwide (21). Rubella still circulates in Zambia as evidenced through the measles case based surveillance program that tests all negative measles cases for rubella (16). Although literature confirms that in most countries where there is no rubella immunization program, most children should have gained natural immunity by age 15 years, there is evidence of at least 5% women of child bearing age still being susceptible (5). This study reveals that the rubella immunity among the female blood donors in Zambia was at 91.9% leaving a susceptible female population of 8.1%, which is within the range determined by the three largest studies in Africa on women of reproductive age that found that 6–16% were susceptible to rubella virus infection (5). This relatively high rate must give concern. All the rubella IgG seronegative participants were between 16 and 33 years of age (within the child bearing age) and this insinuates an increased risk of CRS in this study population.

**Figure 2:**
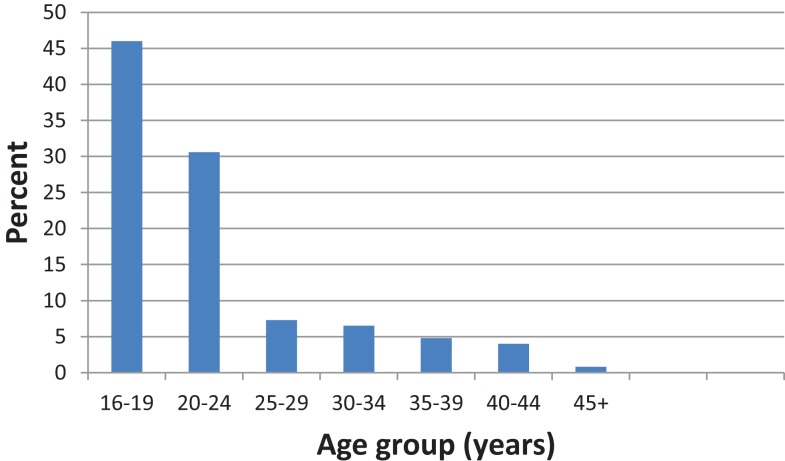
**Age distribution**.

The current study endeavored to determine what demographic and socio-economic factors including age, residency, education, and employment among were associated with rubella seronegativity. There was no significant association between any of these factors and rubella seronegativity (Table 2). This lack of association could be due to the small sample size and the majority of the population (46.0%) being skewed toward the younger age group below 24 years (Figure 2). The lack of association in the current study may partly be due to most participants having similar economic status. The study took place during school days and at this time most blood collections are done at schools rather than community based.

**Table 2:** **Factors associated with rubella seronegativity**.

Factor	IgG total	IgG negative (%)	OR (95% CI)
**Age (years)**
<20	57	6 (10.7)	1.38 (0.71, 2.66)
20+	67	4 (6.0)	1
**Marital status**
Never married	79	9 (11.4)	2.35 (0.82, 6.72)
Currently or once married	44	1 (2.3)	1
**Residence**
Urban	47	6 (12.8)	1.62 (0.84, 3.14)
Rural	76	4 (5.3)	1
**Education level**
No formal school/up to secondary school	63	3 (4.8)	0.61 (0.30, 1.24)
Secondary school completed/college/pre-university or higher	50	6 (12.0)	1
**Current employment**
Government/non-government/self employed/employer	24	3 (12.5)	1.60 (0.50, 5.19)
Not working for pay	19	1 (5.3)	1

It is interesting to note that literature reveals increasing seropositivity with increasing age while others showed advancing age to be associated with reduced risk of rubella (22). However, in the current study, no association was observed between age and rubella seronegativity. Lack of adequate power in our study may explain our finding.

Although our study agrees with some studies such as by Karakoc et al. (23) that show no significant association of the outcome with economic status, low socio-economic status has also been seen to be associated with risk of rubella infection in other studies (24, 25). Our study agrees with authors such as Kombich et al. (25) that found no association between education and rubella infection.

We note that type II error could have been minimum because after the re-analysis for sample size based on the current study proportion of seronegative females of 8.1%, population size of 48,733, and confidence limit of 5%, using statcalc program in epi info version 7, the sample size should have been 114. In this manuscript, we have a sample size of 124; therefore, the sample size in the study is adequate to estimate the proportion of seronegativity.

The study though was limited to the blood donor population who were mostly single; it is an important step in generating baseline seroprevalence data on rubella infection in Zambia. The study did not include most females who were married and in the child bearing period of their lives. Hence, the prevalence of seronegative women to assess the magnitude of the problem in this group of females could not be assessed. It is known that false positivity may occur in persons treated for an immune system disorder, those exposed to parvovirus, those with rheumatoid arthritis or had a transfusion (26). This study did not control for any of these possible confounders except rheumatoid factor during the testing procedure. Though the question as to whether one had had a blood transfusion or received blood products were captured on the blood donor form, it was not collected in this study. However, we believe that the false positives would be minimal. Though no significant association of age with outcome was found in this study it is noted that 8.1% of the study population was still susceptible to rubella infection by age of 16 years. Protection against rubella through natural infection appears inadequate. We recommend the measles vaccine be replaced with measles–rubella vaccine in the under-five immunization program to prevent continued circulation of the virus and a catch up campaign to include older children.

## Conflict of Interest Statement

The authors declare that the research was conducted in the absence of any commercial or financial relationships that could be construed as a potential conflict of interest.

## References

[1] Frey TK Molecular biology of rubella virus. Adv Virus Res (1994) 44:69–160 10.1016/S0065-3527(08)60328-0 7817880 PMC7131582

[2] Dewan P Gupta P. Burden of congenital rubella syndrome (CRS) in India: a systematic review. Indian Pediatr (2012) 49(5):377–99. 10.1007/s13312-012-0087-4 22700664

[3] de Moraes JC Toscano CM de Barros EN Kemp B Lievano F Jacobson S Etiologies of rash and fever illnesses in Campinas, Brazil. J Infect Dis (2011) 204(Suppl 2):S627–36. 10.1093/infdis/jir490 21954258

[4] Centers for Disease Control and Prevention. Achievements in public health: elimination of rubella and congenital rubella syndrome – United States, 1969–2004. Morb Mortal Wkly Rep (2005) 54(11):279–82 Available from: http://www.cdc.gov/mmwr/preview/mmwrhtml/mm5411a5.htm 15788995

[5] Goodson JL Masresha B Dosseh A Byabamazima C Nshimirimana D Cochi S Epidemiology of rubella in Africa in the prevaccine era, 2002-2009. J Infect Dis (2011) 204(Suppl 1):S215–25 10.1093/infdis/jir108 21666164

[6] World Health Organisation. Status Report on Progress Towards Measles and Rubella Elimination. SAGE Working Group on Measles and Rubella (2015). Available from: http://www.who.int/immunization/sage/meetings/2012/november/1_Status_Report_Measles_Rubella_22_Oct.pdf

[7] Otaigbe BE Brown T Esu R Confirmed congenital rubella syndrome. A case report. Niger J Med (2006) 15(4):448–50. 17111737 10.4314/njm.v15i4.37268

[8] Losos J Report of the workgroup on viral diseases. Bull World Health Organ (1998) 76:94. 10063683 PMC2305664

[9] Castillo-Solorzano C Marsigli C Bravo-Alcantara P Flannery B Ruiz Matus C Tambini G Elimination of rubella and congenital rubella syndrome in the Americas. J Infect Dis (2011) 204(Suppl 2): S571–8 10.1093/infdis/jir472 21954249

[10] Hinman A Orenstein W Hart K Preblud S Rational strategy for rubella vaccination. Lancet (1983) 1(8314–5):39–40 10.1016/S0140-6736(83)91572-6 6129376

[11] Freestone DS Programmes for the prevention of rubella during pregnancy by active immunization. Br J Prev Soc Med (1974) 28(4):258–64. 4455345 10.1136/jech.28.4.258 PMC478872

[12] Plotkin SA. Rubella eradication. Vaccine (2001) 19:3311–9. 10.1016/S0264-410X(01)00073-1 11348695

[13] Gilani Z. Population Immunity to Measles and Rubella Viruses in Rural Zambia. Ph.D. thesis, Johns Hopkins University, Baltimore, MD (2013). 219 p.

[14] Al-Sheref F Jefrri OH El-Sayed ZMF Seroprevalence of rubella among pregnant women and young females. Egypt J Med Microbiol (2010) 19:1.

[15] Gomwalk NE Ahmad AA. Prevalence of rubella antibodies on the African continent. Rev Infect Dis (1989) 11(1):116–21. 10.1093/clinids/11.1.116 2783785

[16] Mazaba Liwewe ML Ndumba I Chibumbya J Msiska T Mutambo H Micheelo C Rubella Amongst Suspected Measles Cases Under the EPI Surveillance Program in Zambia (2004–2011). Abstract No. 41.034. 15th ICID. Bangkok (2012). Available from: http://www.xcdsystem.com/icid2012/41.034.html

[17] Mazaba-Liwewe ML Monze M Babaniyi AO Siziya S Phiri D Mulenga J Sero-prevalence of rubella specific immunoglobulin G antibodies and its correlates among blood donors in Zambia. Int Public Health J (2015) 7(4). (in press).

[18] Ministry of Health. Highlights on National Blood Transfusion Status and Trends. Lusaka: Ministry of Health (2013).

[19] University Teaching Hospital. Virology Laboratory Annual Report 2011. Lusaka: Ministry of Health (2011).

[20] Siemens.Enzygnost^®^. Anti-Rubella Virus/IgG Handbook (2011).

[21] Robertson SE Featherstone DA Gacic-Dobo M Hersh BS. Rubella and congenital rubella syndrome: global update. Rev Panam Salud Publica (2003) 14(5):306–15. 10.1590/S1020-49892003001000005 14870758

[22] Cengiz AT Kiyan M Dolapci GI Aysev D Tibet M Serum rubella IgG and IgM antibodies with ELISA in different age groups. Turkish J Infect (1996) 10:249–52.

[23] Karakoc GB Altintas DU Kilinc B Karabay A Mungan NO Yilmaz M Seroprevalence of rubella in school girls and pregnant women. Eur J Epidemiol (2003) 18(1):81–4. 10.1023/A:1022538407514 12705627

[24] Figueiredo CA Afonso AM Curti SP Oliveira MI Souza LT Sato HK Seroprevalence of rubella in urban and rural populations, Guaratinguetá. Rev Assoc Med Bras (2009) 55(2):117–20. 10.1590/S0104-42302009000200011 19488643

[25] Kombich JJ Muchai PC Tukei P Borus PK. Rubella; seroprevalence among primary and pre-primary pupils at Moi’s bridge location, Uasin Gishu district, Kenya. BMC Public Health (2009) 9:269. 10.1186/1471-2458-9-269 19640288 PMC2731100

[26] University of Rochester Medical Center. Health Encyclopedia: Rubella (2015). Available from: http://www.urmc.rochester.edu/encyclopedia/content.aspx?ContentTypeID=167&ContentID=rubella

